# Diphtheria Presenting With Cranial Neuropathy: The Patch Unseen

**DOI:** 10.7759/cureus.101241

**Published:** 2026-01-10

**Authors:** Sonali Ghosh, Shamsul Hoque, Soumodip Saha, Saikat Sadhukhan, Kaushik Ghosh

**Affiliations:** 1 Emergency Medicine and Critical Care, Institute of Post-Graduate Medical Education and Research and Seth Sukhlal Karnani Memorial Hospital, Kolkata, IND; 2 Emergency Medicine, Institute of Post-Graduate Medical Education and Research and Seth Sukhlal Karnani Memorial Hospital, Kolkata, IND; 3 Medicine, Murshidabad Medical College & Hospital, Baharampur, IND

**Keywords:** antitoxin, diphtheria, neuromuscular disorders, polyneuropathy, vaccination

## Abstract

Diphtheria is a vaccine-preventable disease that remains endemic in several developing countries. Neurological complications, such as polyneuropathy, are rare but serious, often mimicking other neuromuscular disorders and leading to delayed diagnosis. Herein, we report the case of a 20-year-old incompletely immunised woman who presented with bilateral ptosis, multiple cranial neuropathies, and mild limb weakness following a prodrome of fever, headache, and peri-orbital pain. Initial investigations revealed albumin-cytologic dissociation in the cerebrospinal fluid, raising suspicion of Guillain-Barré syndrome, for which intravenous immunoglobulin was administered; however, there was no clinical improvement. Myasthenia gravis and botulism were also considered but excluded based on clinical and laboratory findings. Throat swab culture indicated the presence of *Corynebacterium diphtheriae*, confirming the diagnosis of diphtheritic polyneuropathy. The patient was treated with diphtheria antitoxin and erythromycin, resulting in an improvement of bulbar symptoms; nonetheless, ptosis persisted at discharge. Prophylaxis was provided to close contacts, and the patient was scheduled for diphtheria vaccination. This case highlights the significance of considering diphtheria in the differential diagnosis of acute cranial neuropathies in endemic regions and the role of vaccination in preventing such life-threatening complications.

## Introduction

Diphtheria is an acute, toxin-mediated infectious disease caused by *Corynebacterium diphtheria* [1]. Despite the widespread availability of effective vaccination, diphtheria remains a public health concern in developing countries owing to incomplete immunisation, waning immunity, and occasional outbreaks [1,2]. The National Health Profile 2022 indicates that India recorded 1,586 cases and 22 fatalities due to diphtheria in 2020, followed by 3,677 cases and 47 deaths in 2021. Data from the Global Health Observatory repository (2022) reveals a steady increase in diphtheria cases globally, with India, Niger, Indonesia, and Pakistan reporting the highest numbers of infections. The disease primarily affects the upper respiratory tract, producing pharyngeal pseudomembranes and systemic toxicity. Nevertheless, neurological complications are also well-recognised and typically occur two to six weeks after the onset of the primary infection [3,4].

Post-diphtheritic polyneuropathy is one of the most serious complications and may clinically mimic other neuromuscular disorders, such as Guillain-Barré syndrome (GBS), myasthenia gravis (MG), or botulism [5,6]. GBS is characterised by symmetrical cranial neuropathies, bulbar weakness, limb weakness, and occasionally respiratory muscle involvement. In the absence of typical throat findings, albumino-cytologic dissociation in the cerebrospinal fluid often poses diagnostic difficulties [7,8].

Herein, we report the case of an incompletely immunised young woman who presented with bilateral ptosis and multiple cranial nerve palsies. The patient was initially suspected to have GBS or MG, but subsequent microbiological testing confirmed the presence of diphtheria-associated polyneuropathy. This case underscores the importance of considering diphtheria in the differential diagnosis of acute cranial neuropathies in endemic regions [9,10].

## Case presentation

**Figure 1:**
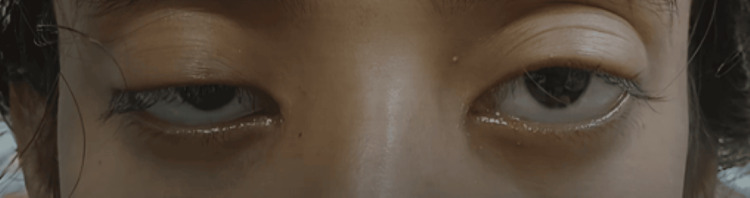
Asymmetric bilateral ptosis (on admission)

 A 20-year-old woman was brought to the emergency department with complaints of slurred speech with nasal intonation, difficulty swallowing, and drooping of both upper eyelids (Figure 1) for three days. She had a low-grade fever three days ago and headache, earache, and peri-orbital throbbing pain nine days ago. She denied any history of respiratory distress, loss of consciousness, deviation of the angle of the mouth, sensory involvement, motor weakness, or bladder or bowel involvement.

**Figure 2:**
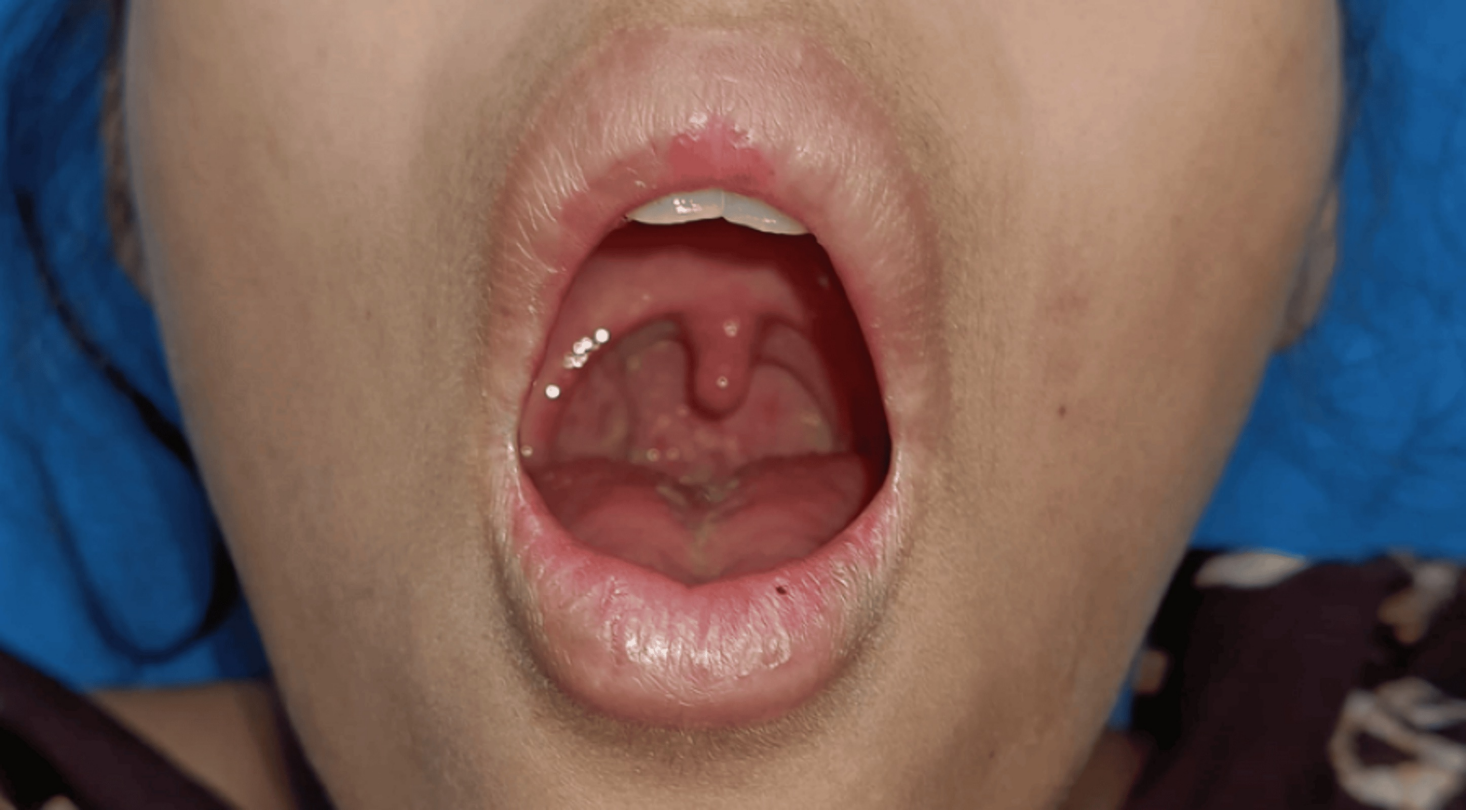
Oral cavity showing normal tonsillar pillar and uvula in the midline

On examination, her vitals were stable (blood pressure: 100/60 mmHg, respiratory rate: 25/min, pulse rate: 100 beats per minute, oxygen saturation: 99% in room air, and temperature: 99.9°F). Pallor, cyanosis, clubbing, oedema, and icterus were absent. No palpable lymphadenopathy was present, and she was alert, conscious, and oriented. There was profound weakness in neck extension, and weakness of the bilateral trapezius and sternocleidomastoid was noted. Cranial nerve examination revealed bilateral mildly dilated pupils that reacted slowly to light, bilateral lateral rectus palsy, bilateral lower motor neuron-type facial nerve palsy, neck weakness, and slightly reduced power in both upper and lower limbs. Oral cavity examination revealed uvula in midline, weakness of tongue and palate movement, and no sign of inflammation (Figure 2). Gag reflex and jaw jerk were absent. Deep tendon reflexes and sensory function were preserved in all four limbs. Other systemic examinations were normal.

Laboratory investigations (Table 1) showed neutrophilic leucocytosis. Liver and renal function tests were normal. The immunological work-up for anti-nuclear antibody (ANA) and ANA profile was negative.

**Table 1:** Summary of laboratory investigations CSF - Cerebrospinal Fluid

Parameter	Patient Value	Reference Range	Unit
Hemoglobin (Hb)	12.1	12.0-15.5	g/dL
Total Leukocyte Count (TLC)	16,270	4000-11,000	/cumm
Neutrophils	92	40-75	%
Platelet Count	200,000	150,000-450,000	/cumm
C-reactive Protein (CRP)	0.9	< 5	mg/L
Procalcitonin	< 0.02	< 0.1	ng/mL
Serum Sodium (Na⁺)	139	135-145	mmol/L
Serum Potassium (K⁺)	4.1	3.5-5.0	mmol/L
Serum Urea	24	10-45	mg/dL
Serum Creatinine	0.8	0.6-1.3	mg/dL
Aspertate Transaminase (AST)	32	< 40	U/L
Alaline Transaminase (ALT)	28	< 40	U/L
Total Bilirubin	0.8	0.2-1.2	mg/dL
Thyroid-Stimulating Hormone (TSH)	2.4	0.4-4.0	µIU/mL
CSF Protein	87	15-45	mg/dL
CSF Cell Count	< 5	0-5	cells/cumm
CSF Glucose	68	40-70	mg/dL

**Figure 3:**
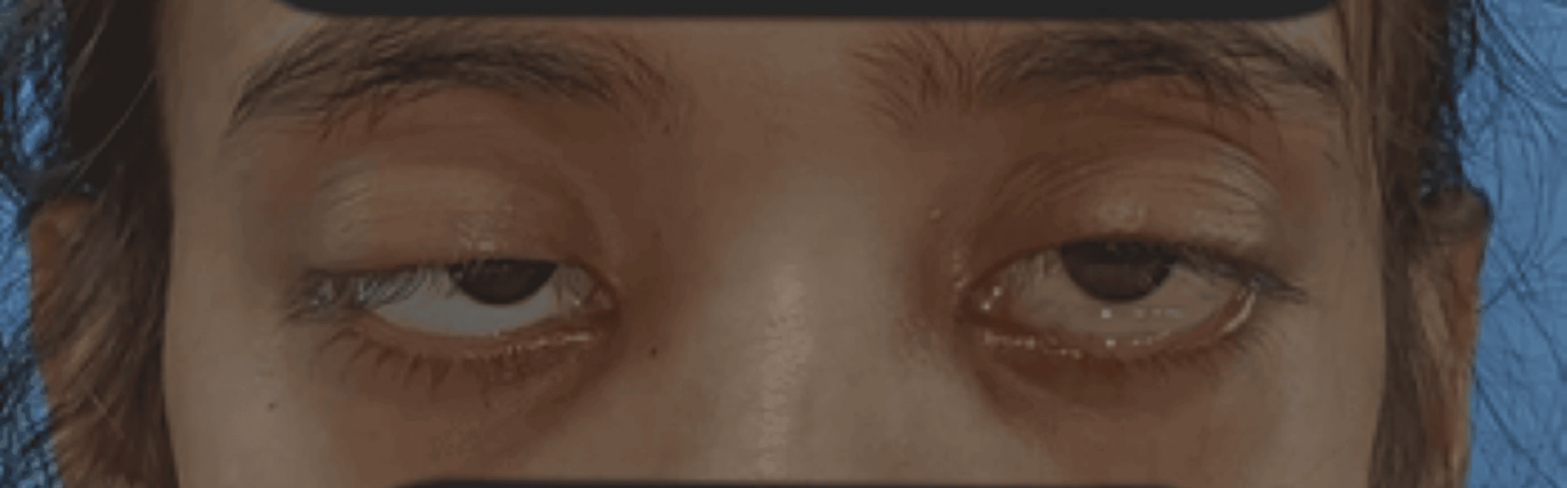
Ptosis persisted but facial nerve functions are mildly improved after treatment with intravenous immunoglobulin

**Figure 4:**
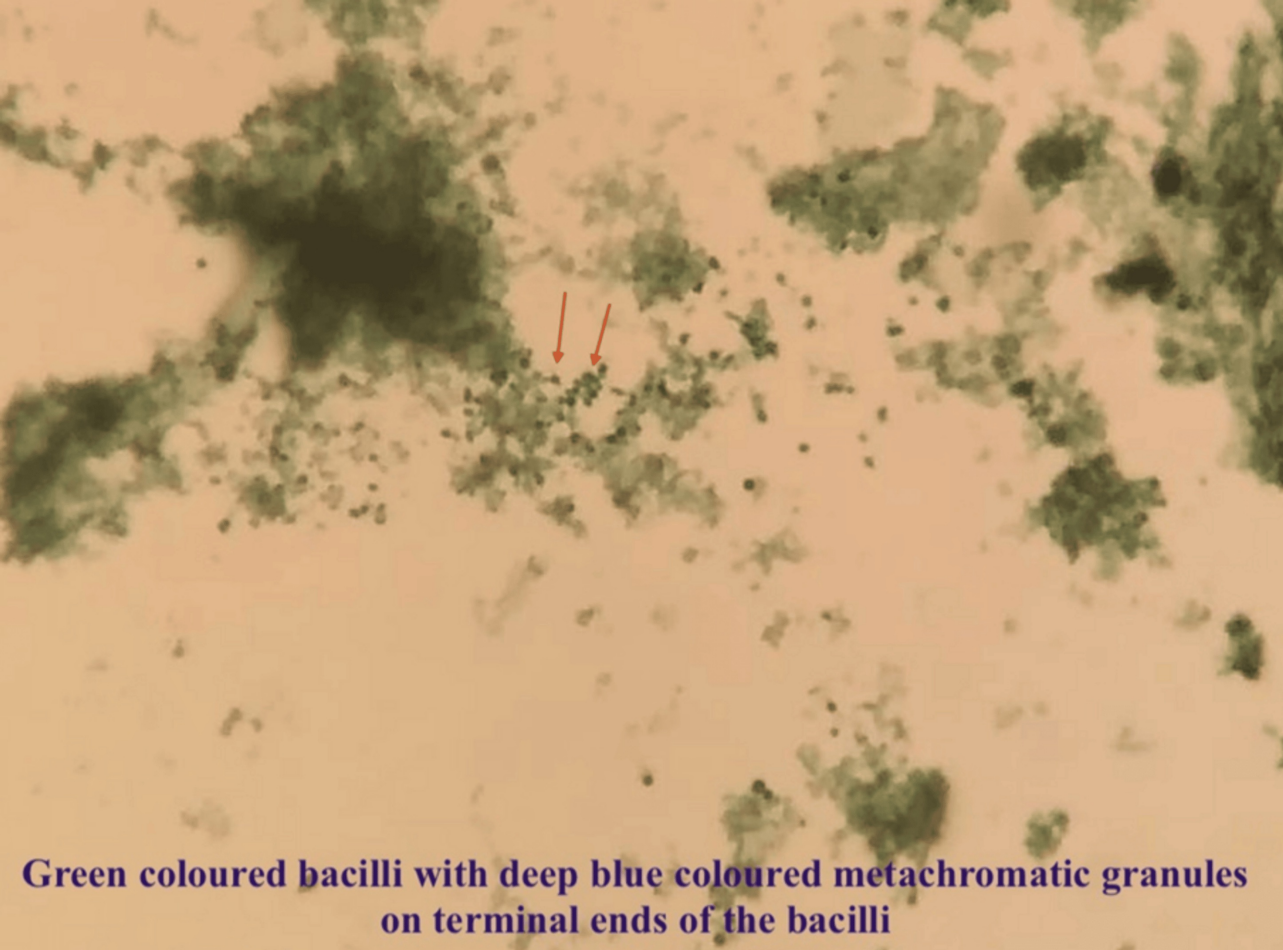
Albert stain showing green-coloured bacilli with metachromatic granules inside the bacilli on microscopy

The bedside ice pack test showed no improvement in ptosis. Nerve conduction study and repetitive nerve stimulation test were normal. Cerebrospinal fluid showed increased protein with a normal cell count. The acetylcholine receptor antibody was negative. Furthermore, resonance imaging of the brain and orbit, along with a venogram, revealed no focal lesions in the cerebral parenchyma or symmetrical optic nerves; however, signal changes were noted. Based on clinical suspicion, intravenous immunoglobulin (0.4 mg/kg/day) was administered for five days, in conjunction with high-grade antibiotics. However, there was only mild improvement in ptosis (Figure 3). The muscle-specific kinase (MuSK) antibody was negative, and subsequently, a throat swab was sent. It showed club-shaped gram-positive bacilli in Gram stain and green-coloured bacilli with one or multiple metachromatic granules inside them (Figure 4). A history of incomplete immunisation was revealed on further questioning.

The patient received one lakh units of diphtheria antitoxin, followed by oral erythromycin for 14 days. Bulbar symptoms improved, but ptosis persisted. Oral azithromycin was given to her close contact (mother) for seven days. Adult diphtheria vaccination was planned for the patient after a month. She was discharged in a haemodynamically stable condition and was followed up at the infectious disease and neuromedicine outpatient departments.

Informed consent was taken from the patient for publication to contribute to medical education and scientific knowledge.

## Discussion

Diphtheria is an acute infectious disease caused by toxigenic strains of *C. diphtheriae*. Despite the availability of vaccination, sporadic cases and outbreaks continue to occur in developing countries, largely because of incomplete immunisation and waning immunity in adults [1,2]. Neurological complications are among the most serious sequelae and may appear two to six weeks after the primary infection [3].

Our patient presented with bilateral ptosis, multiple cranial neuropathies, and mild limb weakness following a prodromal illness. Albumino-cytologic dissociation in cerebrospinal fluid initially raised suspicion of GBS. However, the absence of clinical improvement after intravenous immunoglobulin therapy, combined with the identification of *C. diphtheriae* in a throat swab culture, confirmed the diagnosis of diphtheritic polyneuropathy. This overlap between GBS and diphtheria-associated neuropathy has been described previously, and misdiagnosis is common in the absence of pharyngeal findings [4,5].

The neurological manifestations of diphtheria could be attributed to the systemic absorption of diphtheria toxin, which inhibits protein synthesis and leads to segmental demyelination of peripheral nerves [6]. Cranial nerves are particularly susceptible, with the oculomotor, abducens, facial, glossopharyngeal, and vagus nerves commonly affected [7]. Bulbar weakness can result in dysphagia, nasal regurgitation, and aspiration pneumonia, whereas diaphragmatic involvement may cause respiratory failure [8]. In our patient, bulbar symptoms improved following the administration of diphtheria antitoxin and antibiotic therapy, although ptosis persisted. This finding agrees with earlier reports that recovery of cranial neuropathies may take weeks to months, depending on the extent of axonal injury [9].

Differentiating diphtheritic polyneuropathy from other neuromuscular disorders is crucial for the timely management of the disease. MG is characterised by fatigable weakness, positive acetylcholine receptor or MuSK antibodies, and improvement with the ice pack test, none of which were seen in our patient. Botulism, another differential diagnosis, typically presents with descending paralysis, autonomic dysfunction, and a history of ingestion of contaminated food [10]. However, the absence of gastrointestinal prodrome and the positive throat swab culture excluded this possibility.

The mainstay of treatment for diphtheria is the prompt administration of the antitoxin, which neutralises the circulating toxin but does not reverse the neuronal damage. Antibiotic therapy with erythromycin or penicillin is essential for eradicating the organism and preventing transmission [1]. Chemoprophylaxis for close contacts and vaccination after recovery remain crucial in limiting the spread of the disease and ensuring long-term immunity [2,10].

This case highlights three key points: (i) diphtheria should be considered in the differential diagnosis of acute cranial neuropathies in endemic regions; (ii) early recognition and administration of antitoxin are vital for favourable outcomes; and (iii) strengthening the immunisation programmes remains the most effective strategy to prevent such potentially life-threatening complications.

## Conclusions

Diphtheria continues to pose a diagnostic challenge in regions with incomplete immunisation coverage. Neurological complications, especially polyneuropathy with multiple cranial nerve involvement, can closely mimic conditions such as GBS, MG or botulism. Early recognition, throat swab culture and timely administration of diphtheria antitoxin are essential for patient survival and functional recovery. This case emphasises the significance of maintaining a high index of suspicion for diphtheria in patients presenting with acute cranial neuropathies, particularly in endemic areas, and underscores the pivotal role of immunisation in preventing such life-threatening complications.
